# Underreporting of the 5-year tetanus, diphtheria, pertussis and polio booster vaccination in the Danish Vaccination Register

**DOI:** 10.1186/s12889-020-09816-w

**Published:** 2020-11-10

**Authors:** Sidsel Skou Voss, Ida Glode Helmuth, Camilla Hiul Suppli, Palle Valentiner-Branth

**Affiliations:** 1 European Programme for Intervention Epidemiology Training (EPIET), European Centre for Disease Prevention and Control (ECDC), Stockholm, Sweden; 2 Infectious Disease Epidemiology and Prevention, Statens Serum Institut, 2300 Copenhagen S, Artillerivej 5 Denmark

**Keywords:** The Danish vaccination register, TdaP-IPV, Validation, Underreporting, Immunization, Recall, Reminder

## Abstract

**Background:**

In Denmark, vaccination coverage is measured using the Danish Vaccination Register (DDV). In general, the vaccination coverage is high, but for some vaccinations, the coverage is suboptimal with geographical variation. This study aims to validate the vaccination coverage of the 5-year booster and identify overall reasons for non-vaccination in Copenhagen.

**Methods:**

We validated the coverage of the 5-year tetanus, diphtheria, pertussis and polio booster for children born in 2010 and living in Copenhagen municipality in 2018, an area with low coverage (current estimate: 89%). We identified all children born in 2010 in the Civil Registration System and sent an electronic questionnaire to parents of children without a record of the 5-year booster in the DDV.

**Results:**

Parents of 692 children were contacted and 49% participated. Of those, 186 (55%) reported that the child was vaccinated: 61% by their general practitioner and 34% abroad. The most common reason for non-vaccination was forgetfulness (31%), 26% did not want their child vaccinated and 17% had migrated from abroad and were not aware of the vaccination schedule. Considering only children with documentation for the vaccination, the corrected vaccination coverage was 91%.

**Conclusions:**

We conclude that the coverage of the 5-year booster in Copenhagen is currently underestimated and should be adjusted by 2%. We recommend increased awareness from general practitioners and tailored communication about the vaccination programme targeting immigrants in Denmark.

**Supplementary Information:**

**Supplementary information** accompanies this paper at 10.1186/s12889-020-09816-w.

## Background

The Danish Health Authority recommends immunizing children against ten infectious diseases [1]. The childhood vaccinations are free, and are given by the general practitioner. Parents have to book a vaccination consultation in advance. Migrants are not systematically offered health examination and vaccination on arrival, and consulting their general practitioner for getting childhood vaccinations requires both knowledge of the Danish healthcare system’s organization and services, and language proficiency. Denmark has no vaccination policy for school entry. Surveillance of the vaccination programme is conducted at the Statens Serum Institut (SSI) through the Danish Vaccination Register (DDV). National estimates of vaccination coverage are produced based on data from the DDV and reported to WHO annually. In general, the vaccination coverage is high, but for some vaccinations the coverage is suboptimal and there is geographical variation [2]. Copenhagen is the largest municipality in Denmark and has one of the lowest coverages. The combined tetanus, diphtheria, pertussis and polio booster given at 5 years of age is among the vaccinations with the lowest uptake. In order to raise vaccination coverage, SSI has sent out reminder letters to parents of all children missing one or more vaccinations in the programme at specified ages since 2014 [3] and registration of administered vaccinations has become mandatory.

This study aims to validate the vaccination coverage of the 5-year booster and identify reasons for non-vaccination in Copenhagen.

## Methods

We used the Civil Registration System (CRS) [4] to identify children born in 2010 and living in Copenhagen municipality as of July 2018. In the CRS, all residents in Denmark and people with residence permit are registered with a unique personal identification number. Using the unique personal identifier we linked data from CRS to the DDV and identified children not registered with the 5-year booster in the DDV. An electronic questionnaire, see supplementary material 1, (adapted from Wójcik et al. [5]) was sent to their parents/guardians via ‘e-Boks’, a personal electronic mailbox used to receive mail from the public authorities. The questionnaire included information on the study and a possibility to decline participation. One electronic reminder and one paper version of the questionnaire was sent for non-responders. For parents who still had not responded, we attempted to contact them by telephone. The study fell under the general agreement for non-interventional database studies between the Danish Data Protection Agency and Statens Serum Institut, and ethics approval of the survey (questionnaires and interviews) was not necessary according to Danish legislation.

The questionnaire collected information on whether the child had received the 5-year booster including where and when, and if applicable, reasons for the child not being vaccinated. A vaccination was considered documented if the parents could provide a date of vaccination from written documentation or if the vaccination had been confirmed by the parents contacting their general practitioner.

We calculated corrected vaccination coverages by adding the proportion of vaccinated in the questionnaire study to the vaccination coverage in the DDV using the following equation as described by Wójcik et al. [5],

1 $$ \mathrm{r}+\left(1-\mathrm{r}\right)\ast \mathrm{q}, $$

where r is the vaccination coverage in the DDV, q is the estimated proportion of vaccinated in the questionnaire study.

The corrected vaccination coverages were calculated under two different assumptions; 1. non-participants were not vaccinated, 2. non-participants were vaccinated at the same rate as participants. For each of these assumptions two further vaccination coverages were estimated; one including all children who answered ‘yes’ to vaccination, and one including only children with documented vaccination. In total, four vaccination coverages were estimated from a least conservative estimate to a most conservative estimate. We calculated 95% confidence intervals for q on the log (odds) scale and transformed back to a proportion scale. The upper and lower confidence limits for q were used in the equation (as q) to estimate the confidence intervals for the corrected vaccination coverage.

Data from the questionnaire were analysed in STATA (version 14 StataCorp Texas). Categorical data were compared using a chi^2^ test.

## Results

**Fig. 1:**
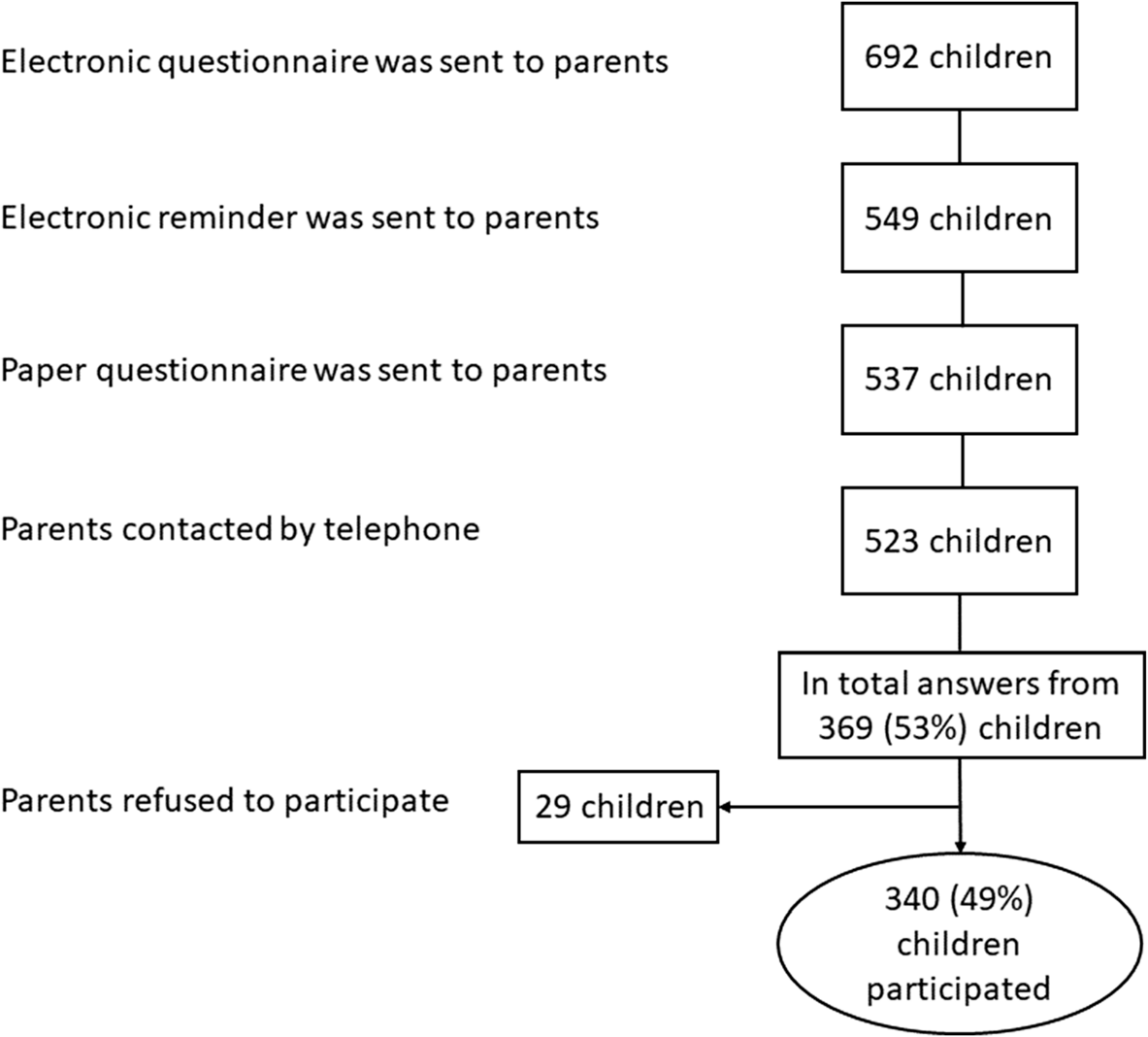
Flow chart of inclusion of children in the study

We identified 6039 children born in 2010 who lived in Copenhagen as of July 2018. Of those, 89% were registered with the 5-year booster in the DDV. Parents of 692 (11%), children were invited to participate and 49% of those responded, Fig. 1.

The distribution between boys and girls in the study population was equal (50% vs. 50%), but there was a small non-significant preponderance of boys among study participants (53% vs 47%), Table 1. The distribution of participants with Danish origin, second generation immigrants and immigrants, respectively, reflects the distribution among all children not registered with the 5 years booster. Compared to the general population of 10 year old children in Copenhagen in 2018 (22%) [6] the group of second generation immigrants and immigrants was overrepresented in this study population (39%), showing that Danish born children had a higher vaccination coverage. Participants and non-participants had comparable distributions across all variables except residential district, but by removing the outlier, Vesterbro-Kgs Enghave, this difference was no longer significant.

**Table 1:** Characteristics of 692 children born in 2010, not registered with the 5-year booster

	All	Participants	Non-participants	P
**Sex**				
Male	347 (50%)	179 (53%)	168 (48%)	0.196
Female	345 (50%)	161 (47%)	184 (52%)	
**Origin**				
Danish	426 (62%)	217(64%)	209 (59%)	0.432
Second generation immigrants	121 (17%)	54 (16%)	67 (19%)	
Immigrants	145 (22%)	69 (20%)	76 (22%)	
**District**				
Amager	153 (22%)	76 (22%)	77 (22%)	0.014
Østerbro	119 (17%)	67 (18%)	52 (15%)	
Brønshøj-Husum	79 (11%)	30 (9%)	49 (14%)	
Vesterbro - Kgs. Enghave	83 (12%)	54 (16%)	29 (8%)	
Nørrebro	75 (11%)	35 (10%)	40 (11%)	
Bispebjerg	59 (9%)	24 (7%)	35 (10%)	
Valby	51 (7%)	20 (6%)	31 (9%)	
København K	37 (5%)	19 (6%)	18 (5%)	
Vanløse	28 (4%)	13 (4%)	15 (4%)	
Unknown	8 (1%)	2 (1%)	6 (2%)	

In total, 186 children (55%) were vaccinated with the 5-year booster according to their parents, Table 2, and 128 (69%) had documentation, either as written documentation (*n* = 81, 44%) or had the vaccination confirmed at the general practitioner (*n* = 47, 25%). The majority of the children were vaccinated at their general practitioner (61%) or abroad (34%). The most common cause for non-vaccination was forgetfulness (31%). Parents of 28 children (26%) had actively declined vaccination and 17% were not aware of the Danish vaccination programme after immigration to Denmark.

**Table 2:** Answers from 340 children living in Copenhagen born in 2010, not registered with 5-year booster

	5-year booster
**Vaccination**	
Yes	186 (55%)
No	109 (32%)
Don’t know	45 (13%)
**Vaccinated where**	
General practitioner	114 (61%)
Abroad	63 (34%)
Pediatrician	3 (2%)
Hospital	2 (1%)
Don’t know	2 (1%)
At home^a^	1 (1%)
Travel clinic	1 (1%)
**Why not vaccinated**	
Forgot	34 (31%)
Do not want vaccination	28 (26%)
Immigrated and not aware^b^	19 (17%)
Previous adverse events from vaccinations	5 (5%)
Child cannot tolerate according to the doctor	3 (3%)
Child sick at vaccination, not rescheduled	3 (3%)
Did not have time	2 (2%)
Other	15 (14%)

^a^ The child was vaccinated by the father, who is a physician

^b^ Moved from abroad and not aware of the Danish Vaccination Schedule

Table 3 shows four calculations of corrected vaccination coverage of the 5-year booster. The most conservative estimates of 91 and 92% were calculated under the assumption that non-participants were not vaccinated, and children with documented vaccination as well as all children whose parents answered ‘yes’ to vaccination were included in the calculation. Similarly, the estimates of 93 and 95% were calculated considering non-participants vaccinated at the same rate as respondents.

**Table 3:** Corrected vaccination coverages for children living in Copenhagen born in 2010

	Vaccination coverage considering non-participants as not vaccinated			
	Vaccination coverage in the study, % (95% CI)		Corrected vaccination coverage among children in Copenhagen born in 2010, % (95% CI)	
Documented vaccination^a^	128/692	18% (16, 22%)	0.89^b^ + (1–0.89) × 0.18	91% (90, 91%)
Answered ‘yes’ to vaccination	186/692	27% (23, 31%)	0.89^b^ + (1–0.89) × 0.27	92% (91, 92%)
	Vaccination coverage considering non-participants as vaccinated at the same rate as participants			
	Vaccination coverage in the study, % (95% CI)		Corrected vaccination coverage among children in Copenhagen born in 2010, % (95% CI)	
Documented vaccination^a^	128/340	38% (31, 46%)	0.89^b^ + (1–0.89) × 0.38	93% (92, 94%)
Answered ‘yes’ to vaccination	186/340	55% (46, 65%)	0.89^b^ + (1–0.89) × 0.55	95% (94, 96%)

^a^ A vaccination was considered documented if the parents could provide a date of vaccination from a vaccination card or equivalent written documentation or if the parents had the vaccination confirmed by their general practitioner

^b^ Vaccination coverage in the DDV = 5347/6039 (89%)

## Discussion

For children living in Copenhagen as of July 2018, who were born in 2010, we found that the coverage of the 5-year booster can be adjusted by 2–6 percentage points. All though these estimates are based on a single birth cohort in a selected geographical area, Denmark will now adjust the official country estimate reported to WHO with the most conservative estimate of a 2% increase. It could be argued that it is reasonable to adjust the coverage with the least conservative estimate (6%), since we found the participants in the study comparable to the non-participants on several variables. However, from a public health perspective it is important not to risk overestimation of the coverage. Up until now, Denmark has adjusted the vaccination coverage for the 5-year booster with 3 percentage points based on a study from 2013 that also validated the completeness of the DDV with the same method on a national level [5]. Since 2013, Denmark has implemented several interventions in order to raise the vaccination coverage and ensure correct registration of vaccinations in the DDV. Reminder letters are being sent out to parents of children missing one or more vaccinations [3] and registration of given vaccinations directly in the DDV became mandatory in 2015.

Our study implies that the completeness of the DDV has increased. The proportion of children who were vaccinated but not registered was 55%, which is a lower percentage than found by the previous Danish study from 2013 using the same method, and where this proportion was 70% [5]. It is a similar proportion as found by another Danish study from 2017 that validated the completeness of the registered MMR 1 (Measles, Mumps, and Rubella) coverage in a single region of Denmark. This study concluded that the registered vaccination coverage could be adjusted in 55% of children according to medical records [7]. The mandatory registration in the DDV was implemented in November 2015 and therefore only affects a subpopulation of the current study. If the full study population had been included in this intervention, we might have found an even lower proportion of children without correct registration of vaccination. Failure to register vaccinations correctly is still an important reason for children appearing to be non-vaccinated, as 61% of children who were vaccinated but not registered as such, were already vaccinated at their general practitioner. It is possible for doctors and citizens to access the registered information in the DDV and to register vaccinations in the DDV and there might be a need for an increased awareness about this opportunity. Even though registration of given vaccinations is now mandatory, this does not apply to vaccinations given abroad or prior to the legislation. Targeted information to immigrants about the Danish vaccination programme and the DDV is warranted. In total, 34% of the children in this study who were vaccinated, but not registered, were vaccinated abroad and 17% of parents, whose child was not vaccinated, indicated that they had moved to Denmark from abroad and was not aware of the Danish vaccination schedule.

Our study population of children born in 2010 was included in the reminder letter population and had received a reminder in case of missing vaccinations at 6 ½ years of age. The decrease in proportion of forgetful parents from 37% in the 2013 study to 31% in the present study is surprisingly small in this regard, as it has previously been shown by Suppli et al. in a study from 2016, that the reminder letters have had a positive effect on the vaccination coverage [8]. The authors found that the reminders were particular effective on the coverage of the 5-year booster. International studies have also shown that reminding parents of vaccinations is an effective way of raising vaccination coverage [9] A Cochrane Review from 2018 found that reminders improve receipt of childhood vaccinations with 22% [10]. In order to further target forgetful parents, Denmark subsequently introduced pre-vaccination recall in 2019 [11].

Parents not wanting their child vaccinated was the second most common reason for non-vaccination (*n* = 28, 26%). Vaccine hesitancy is defined by WHO’s immunisation SAGE (Strategic Advisory Group of Experts) as ‘delay in acceptance or refusal of vaccinations despite availability of vaccine services’ [12]. Although our study includes a small number of children, the finding that 26% of parents of non-vaccinated children refused vaccination could be explained by the fact that we only studied children in Copenhagen. As described by Olive et al., major metropolitan areas have been associated with high rates of anti-vaccine activities [13]. Several studies in Europe and the US have shown the same association of lower vaccination rates among urban children providing pockets of poor coverage, while other European studies, has identified rural habitation as a risk factor for non-vaccination [14, 15]. Leask et al. found that the parental group defined as ‘refuser’ of all vaccinations represents less than 2% [16] and if we assume the proportion of parents not wanting vaccination in this study is the same among non-participants, then the total proportion of vaccine refusing parents in Copenhagen is about 1%. We do not expect this to have a significant impact on the effect of the vaccination programme on a population level. This result furthermore highlights the importance of validating and examining the root causes behind a low vaccination coverage estimate. It is important that the interventions aimed at increasing vaccination coverage, that countries might consider to implement, target the actual problem.

Limitations of this study include a response rate of only 49%, which could compromise the interpretation and the generalizability of our results, but participants and non-participants were comparable on factors like sex and ethnicity. Furthermore, the study only included subjects living in Copenhagen and as mentioned above this is a limitation in regards to the generalizability of our results. A possible limitation due to misregistrations in the DDV by parents/guardians is considered very limited, as registrations in the DDV done by parents/guardians, and not validated by a doctor, counts for less than 1% of all registrations.

The strengths of our study are the access to high-quality registers with information on individual level that allowed us to contact parents of all children in our chosen cohort as opposed to a mere sample. Furthermore, this allowed us to compare participants and non-participants with regards to significant sociodemographic variables.

## Conclusions

The coverage of the 5-year booster in Denmark is currently underestimated and coverage estimates for Copenhagen could be adjusted by at least two percentage points. Despite reminder letters, forgetfulness is still the most common reason for missing vaccinations. Denmark has subsequently introduced pre-vaccination recalls. We recommend increased awareness from general practitioners and tailored communication about the vaccination programme targeting immigrants to Denmark.

## Supplementary Information

**Additional file 1:** **Supplementary Material 1.** Questionnaire. Paper version of the questionnaire used in this study.

## Data Availability

The datasets generated and analysed during the current study are not publicly available due to Danish law as they contain information that could compromise research participant privacy, but are available in aggregated form from the corresponding author on reasonable request.

## Abbreviations

CRS: Civil Registration System
DDV: Danish Vaccination Register
IPV: Inactivated Polio Vaccine
SSI: Statens Serum Institut
Tdap: Tetanus, diphtheria and acellular pertussis
WHO: World Health Organization
