# Supplementary material for: Underreporting of the 5-year tetanus, diphtheria, pertussis and polio booster vaccination in the Danish Vaccination Register
Source: BMC Public Health. 2020 Nov 10;20:1681. doi: 10.1186/s12889-020-09816-w (PMC7654036; doi:10.1186/s12889-020-09816-w)
Supplement: Supplementary file 1 — Additional file 1: Supplementary Material 1. Questionnaire. Paper version of the questionnaire used in this study. [file 12889_2020_9816_MOESM1_ESM.pdf]

Supplementary material 1

STATENS SERUM INSTITUT

Questionnaire

The questionnaire regards your child born in 2010. Please write your child's name and cpr-number:

----

1. This survey addresses vaccines against diphtheria, tetanus, pertussis, polio and H. influenzae (D-T-aP-Hib-IPV and T-d-aP-IPV booster) and measles, mumps, and rubella vaccines (MMR). The abbreviations will be used in the following.
(State one answer only)

- ❑ Start the survey - Go to 3.
- ❑ I do not wish to participate in the survey - Go to 2.
- ❑ I would like to answer the questionnaire by telephone (please enter your telephone number and the time of day you wish to be contacted)

_ _

2. The reason I do not wish to participate is as follows:

_ _

_ _

3. Has your child been given the first D-T-aP-Hib-IPV vaccine, which is usually given at 3 months of age?
(State one answer only)

- ❑ Yes
- ❑ No - Go to 6.
- ❑ Do not know - Go to 7.

4. Where did your child get the first D-T-aP-Hib-IPV vaccine?

(State one answer only)

- ❑ General practitioner
- ❑ Pediatrician
- ❑ Hospital
- ❑ Travel vaccination clinic
- ❑ Abroad

Other location (please write where below)

_ _

5. Is the information you provided about the first D-T-aP-Hib-IPV vaccine from your child's vaccination card or other written documentation?
(State one answer only)

- ❑ Yes - Go to 7.
- ❑ No - Go to 7.

6. Why did your child not get the first D-T-aP-Hib-IPV vaccine?

(State one answer only)

- ❑ I forgot
- ❑ My child cannot tolerate the vaccine according to the doctor
- ❑ I do not want my child vaccinated
- ❑ I have not had time to have my child vaccinated
- ❑ My child was sick when it was time for vaccination and I did not reschedule a new appoinment

Other reason (please write the reason below)

_ _

7. Has your child been given the second D-T-aP-Hib-IPV vaccine, which is usually given at 5 months of age?
(State one answer only)

- ❑ Yes
- ❑ No - Go to 10.
- ❑ Do not know - Go to 11.

8. Where did your child get the second D-T-aP-Hib-IPV vaccine?
(State one answer only)

- ❑ General practitioner
- ❑ Pediatrician
- ❑ Hospital
- ❑ Travel vaccination clinic
- ❑ Abroad

Other location (please write where below)

_ _

9. Is the information you provided about the second D-T-aP-Hib-IPV vaccine from your child's vaccination card or other written documentation?
(State one answer only)

- ❑ Yes - Go to 11.
- ❑ No - Go to 11.

10. Why did your child not get the second D-T-aP-Hib-IPV vaccine?
(State one answer only)

- ❑ I forgot
- ❑ My child cannot tolerate the vaccine according to the doctor
- ❑ I do not want my child vaccinated
- ❑ I have not had time to have my child vaccinated

- ☐ My child was sick when it was time for vaccination and I did not reschedule a new appoinment

Other reason (please write the reason below)

_ _

11. Has your child been given the third D-T-aP-Hib-IPV vaccine, which is usually given at 12 months of age?
(State one answer only)

- ☐ Yes
- ☐ No - Go to 14.
- ☐ Do not know - Go to 15.

12. Where did your child get the third D-T-aP-Hib-IPV vaccine?
(State one answer only)

- ☐ General practitioner
- ☐ Pediatrician
- ☐ Hospital
- ☐ Travel Vaccination Clinic
- ☐ Abroad

Other location (please write where below)

_ _

13. Is the information you provided about the third D-T-aP-Hib-IPV vaccine from your child's vaccination card or other written documentation?

(State one answer only)

- ☐ Yes - Go to 15.
- ☐ No - Go to 15.

14. Why did your child not get the third D-T-aP-Hib-IPV vaccine?

(State one answer only)

- ❑ I forgot
- ❑ My child cannot tolerate the vaccine according to the doctor
- ❑ I do not want my child vaccinated
- ❑ I have not had time to have my child vaccinated
- ❑ My child was sick when it was time for vaccination and I did not reschedule a new appoinment

Other reason (please write the reason below)

_ _

15. Has your child been given the first MMR vaccine, which is usually given at 15 months of age?

(State one answer only)

- ❑ Yes
- ❑ No - Go to 18.
- ❑ Do not know - Go to 19.

16. Where did your child get the first MMR vaccine?

(State one answer only)

- ❑ General practitioner
- ❑ Pediatrician
- ❑ Hospital
- ❑ Travel vaccination clinic
- ❑ Abroad

Other location (please write where below)

_ _

17. Is the information you provided about the MMR vaccine from your child's vaccination card or other written documentation?

(State one answer only)

- ❑ Yes - Go to 19.
- ❑ No - Go to 19.

18. Why did your child not get the first MMR vaccine?

(State one answer only)

- ❑ I forgot
- ❑ My child cannot tolerate the vaccine according to the doctor
- ❑ I do not want my child vaccinated
- ❑ I have not had time to have my child vaccinated
- ❑ My child was sick when it was time for vaccination and I did not reschedule a new appoinment

Other reason (please write the reason below)

_ _

19. Has your child been given the second MMR vaccine, which is usually given at 4 years of age?

(State one answer only)

- ❑ Yes
- ❑ No - Go to 22.
- ❑ Do not know - Go to 23.

20. Where did your child get the second MMR vaccine?

(State one answer only)

- ❑ General practitioner
- ❑ Pediatrician

- ❑ Hospital
- ❑ Travel Vaccination Clinic
- ❑ Abroad

Other location (please write where below)

_ _

21. Is the information you provided about the second MMR vaccine from your child's vaccination card or other written documentation?

(State one answer only)

- ❑ Yes - Go to 23.
- ❑ Yes - Go to 23.

22. Why did your child not get the second MMR vaccine?

(State one answer only)

- ❑ I forgot
- ❑ My child cannot tolerate the vaccine according to the doctor
- ❑ I do not want my child vaccinated
- ❑ I have not had time to have my child vaccinated
- ❑ My child was sick when it was time for vaccination and I did not reschedule a new appoinment

Other reason (please write the reason below)

_ _

23. Has your child been given the T-d-aP-IPV booster vaccine, which is usually given at 5 years of age?

(State one answer only)

- ❑ Yes
- ❑ No - Go to 27.

24. Where did your child get the T-d-aP-IPV booster vaccine?

(State one answer only)

- ❑ General practitioner
- ❑ Pediatrician
- ❑ Hospital
- ❑ Travel vaccination clinic
- ❑ Abroad

Other location (please write where below)

_ _

- ❑ Do not know - Go to 28.

25. Is the information you provided about the T-d-aP-IPV booster vaccine from your child's vaccination card or other written documentation?

(State one answer only)

- ❑ Yes - Go to 26.
- ❑ No - Go to 28.

26. Please enter the date of vaccination (dd-mm-yyyy)

_ _- Go to 28.

27. Why did your child not get the T-d-aP-IPV booster vaccine?

(State one answer only)

- ☐ I forgot
- ☐ My child cannot tolerate the vaccine according to the doctor
- ☐ I do not want my child vaccinated
- ☐ I have not had time to have my child vaccinated
- ☐ My child was sick when it was time for vaccination and I did not reschedule a new appoinment

Other reason (please write the reason below)

_ _

28. If you at least once have answered that you do not want your child to be vaccinated. Which of the following statements are in accordannce with your attitude towards vaccination.
You can choose more than one answer.

(Multiple answers allowed)

- ☐ "Vaccination is not necessary as the disease vaccinated against does not exist in Denmark anymore"
- ☐ "Vaccination is not necessary as the disease vaccinated against is not dangerous"
- ☐ "It is better for the body to get the disease vaccinated against naturally than to be vaccinated"
- ☐ "The immune system / body cannot tolerate being vaccinated against many diseases at once"
- ☐ "Vaccines contain additives that can harm my child"
- ☐ "I do not trust my doctor's recommendation regarding vaccination"
- ☐ "I do not trust the pharmaceutical industry"
- ☐ "I do not trust the authorities that recommend vaccination"

Other reason (please write the reason below)

_ _

29. Here you can list other relevant information about your child and the vaccinations he or she has or has not received.

_ _

_ _

30. May we possibly call you if we have elaborate questions?

(State one answer only)

❑ No

Yes (please enter your telephone number)

_ _

31. Thank you very much for your help.
